# The Interplay of Sports and Nutrition in Neurological Health and Recovery

**DOI:** 10.3390/jcm13072065

**Published:** 2024-04-02

**Authors:** Vicente Javier Clemente-Suárez, Laura Redondo-Flórez, Ana Isabel Beltrán-Velasco, Pedro Belinchón-deMiguel, Domingo Jesús Ramos-Campo, Agustín Curiel-Regueros, Alexandra Martín-Rodríguez, José Francisco Tornero-Aguilera

**Affiliations:** 1 Faculty of Sports Sciences, Universidad Europea de Madrid, Tajo Street, s/n, 28670 Madrid, Spain; vctxente@yahoo.es (V.J.C.-S.); curielagus@gmail.com (A.C.-R.); josefrancisco.tornero@universidadeuropea.es (J.F.T.-A.); 2 Grupo de Investigación en Cultura, Educación y Sociedad, Universidad de la Costa, Barranquilla 080002, Colombia; 3 Department of Health Sciences, Faculty of Biomedical and Health Sciences, Universidad Europea de Madrid, C/Tajo s/n, Villaviciosa de Odón, 28670 Madrid, Spain; lauraredondo_1@hotmail.com; 4 Psychology Department, Faculty of Life and Natural Sciences, Nebrija University, 28240 Madrid, Spain; abeltranv@nebrija.es; 5 Department of Nursing and Nutrition, Faculty of Biomedical and Health Sciences, Universidad Europea de Madrid, Villaviciosa de Odón, 28670 Madrid, Spain; pedro.belinchon@universidadeuropea.es; 6 LFE Research Group, Department of Health and Human Performance, Faculty of Physical Activity and Sport Science-INEF, Universidad Politécnica de Madrid, 28040 Madrid, Spain; domingojesusramos@gmail.com

**Keywords:** neurology, neurological health, physical activity, dietary practices, prevention, treatment, rehabilitation, neuroimaging

## Abstract

This comprehensive review explores the dynamic relationship between sports, nutrition, and neurological health. Focusing on recent clinical advancements, it examines how physical activity and dietary practices influence the prevention, treatment, and rehabilitation of various neurological conditions. The review highlights the role of neuroimaging in understanding these interactions, discusses emerging technologies in neurotherapeutic interventions, and evaluates the efficacy of sports and nutritional strategies in enhancing neurological recovery. This synthesis of current knowledge aims to provide a deeper understanding of how lifestyle factors can be integrated into clinical practices to improve neurological outcomes.

## 1. Introduction

The intricate nexus of sports, nutrition, and neurological health has emerged as a focal point of scientific inquiry, reflecting a burgeoning interest in how lifestyle factors contribute to the neurobiological landscape. This body of work aims to dissect the complex interactions between physical activity, dietary patterns, and their cumulative effects on neurological conditions, spanning prevention, treatment, and rehabilitation. The investigation pivots on elucidating the biological mechanisms underpinning the impact of these lifestyle choices on neurological well-being, undergirded by advancements in clinical research. Special emphasis is placed on the utility of neuroimaging techniques, which have proven indispensable in mapping the physiological correlates of dietary and exercise interventions on the brain, alongside a critical assessment of cutting-edge neurotherapeutic interventions. Thus, we aim to illuminate the biological pathways through which select nutrients and dietary regimes modulate neural function, foster neuroplasticity, and influence cognitive performance, particularly among individuals engaged in regular physical exertion. This endeavor seeks to establish a vital link between nutritional science and neurology, propelling forward evidence-based dietary recommendations that promise to bolster neurological health and aid in the recovery of athletes and the physically active at large.

The thrust of this exploration extends to the exploration of how dietary interventions can mitigate neuroinflammation, enhance synaptic plasticity, and augment cognitive resilience, drawing upon recent findings that delineate the role of specific macro- and micronutrients in neuroprotection and cognitive function enhancement. Additionally, the role of physical exercise in modulating neurotrophic factors, improving cerebral blood flow, and stimulating hippocampal neurogenesis offers promising avenues for neurorehabilitation strategies. By integrating insights from nutritional biochemistry, exercise physiology, and neurology, this analysis sheds light on the symbiotic relationship between diet, physical activity, and brain health, thereby contributing to the development of holistic interventions tailored to support neurological resilience and recovery.

### Methodology of Search

To meticulously address the objectives of this investigation, a comprehensive literature review methodology was meticulously designed and implemented, emphasizing the integration and critical analysis of both primary and secondary sources. This encompassed a thorough examination of scholarly articles, along with a structured search across key databases and bibliographic indices, ensuring a robust foundation for the review. The methodology was developed to reflect best practices within the field, drawing inspiration from similar studies noted in the literature [1,2,3].

The literature search strategy was anchored in a systematic approach to identify relevant studies, employing a combination of MeSH (Medical Subject Headings) terms and free-text keywords to capture the multifaceted relationship between sports, nutrition, and neurological health. The databases queried included MedLine (PubMed), Cochrane Library (Wiley), Embase, and CinAhl, recognized for their extensive coverage of medical and health sciences literature. The search terms were carefully selected to encompass a broad spectrum of concepts within the realms of sports science, nutritional biochemistry, neurology, and cognitive science, including but not limited to “sports nutrition”, “neurological health”, “cognitive function”, “neuroplasticity”, and “dietary interventions”.

The review was primarily focused on literature published within a specific timeframe, from 1 January 2022 to 15 January 2024. This period was chosen to ensure the inclusion of the most recent and relevant findings in the fields under study, while also considering pivotal earlier works that provide essential context and foundational knowledge. To facilitate a comprehensive analysis, studies published in both English and Spanish were included, recognizing the valuable contributions from diverse linguistic and cultural contexts to the global understanding of the topic.

The inclusion criteria were defined with precision to encompass studies that adhered to rigorous scientific methodological standards, specifically those offering valuable insights into the nexus of sports nutrition and neurological health. Criteria for inclusion explicitly targeted research articles that presented empirical findings, review articles providing critical analyses, and meta-analyses summarizing existing evidence. Exclusion criteria were delineated to omit publications outside the specified timeframe, as well as those not meeting the thematic scope of this review. This included, but was not limited to, gray literature, opinion pieces, and studies lacking in empirical evidence or methodological rigor.

Following the initial search, articles were screened based on their titles and abstracts to ascertain relevance to the study’s objectives. This preliminary screening was followed by a detailed review of the full texts, conducted by multiple authors to ensure a balanced and comprehensive evaluation of each study’s contribution to the field. The selection process was characterized by a collaborative approach, with discussions among the authors to reach a consensus on the inclusion of each piece, thereby ensuring that the analysis was informed by a diverse and pertinent body of literature.

## 2. The Role of Sports and Nutrition in Neurological Health

Currently, we know that different factors can have an impact on the appearance and development of Nervous System (NS) pathologies. Neurological pathologies are a group of diseases with a high prevalence worldwide, with a ratio of 1:3 people affected by a neurological disorder. In addition, it is known that they are the second cause of death in the world, as well as the first cause of disability [4]. Within this group, we find some common pathologies, such as Traumatic Brain Injury (TBI), cerebrovascular accident (CVA); neurodegenerative diseases such as Alzheimer’s Disease (AD), Parkinson’s Disease (PD), Huntington’s Disease (HD), Amyotrophic Lateral Sclerosis (ALS); autoimmune and demyelinating diseases such as multiple sclerosis (MS); chronic pain, among others [5,6]. We can also find other less frequent pathologies such as Erb–Duchenne and Dejerine-Klumpke paralysis, trigeminal neuralgia or (neuromyelitis optica spectrum disorders (NMOSD), among others [7].

Their high prevalence in the population, as well as their personal, family and social repercussions, makes these diseases a public health problem in developed countries. Clinically, patients usually present alterations in the level of consciousness, as well as disabilities that prevent these individuals from being able to feed themselves. However, each pathology presents differentiated symptomatology, although alterations in the SN may be common in some of them [8]. In this sense, thanks to the studies recently carried out with a molecular approach, they have been able to determine that nutrition is closely related to the correct functioning in the communication of the bidirectional microbiota–gut–brain axis [9]. Nowadays, metagenomics techniques allow for sequencing of genetic material, identifying phylogenetic relationships through certain markers, which in this case is usually the 16S rDNA gene (16S ribosomal RNA [10]). Briefly, we know that an adult person has between 600 and 1000 species of microorganisms in his intestinal microbiota, the Gram-positive *Firmicutes* (approx. 60%) and the Gram-negative *Bacteroidetes* (approx. 25%) being the most abundant [11].

The study of the impact of nutrition on different diseases has currently become an area of growing interest for the scientific and health community. This has led to an increase in research along this line, with the aim of identifying biomarkers that can regulate the symptomatology of these diseases. In the case of neurological diseases, previous studies have shown the relationship between nutrition and the functioning of the CNS [12]. Specifically, the CNS is involved in the regulation of electrolytes, as well as glucose homeostasis, which modulate the sensations of thirst and hunger, so these basic physiological functions will be altered to different degrees. This depends mainly on the severity of the injury, its location, or the acute or chronic impact of the injury [13].

On the other hand, the nutritional profile of patients with neurological diseases presents differences. For example, in traumatic neurological injury such as TBI, hypermetabolism occurs, which will be regulated by body temperature and administered sedation [14]. In this case, the hyperproteic enteral diet is necessary to avoid bacterial overgrowth, bacterial translocation, enterocyte atrophy and decreased defenses in the digestive system [15,16]. A study by Nicholson et al. in 2019 was able to show significant differences in the gut microbiota of these patients only 2 h after TBI, with a decrease in α-diversity. The most abundant phyla were *Firmicutes*, *Bacteroidetes*, *Verrucomicrobia*, *Proteobacteria*, *Cyanobacteria*, *Tenericutes* and *Deferribactes*. The *Firmicutes*/*Bacteroidetes* ratio decreased significantly 1 day after TBI [17].

In stroke, the patient’s metabolic status worsens in the days following the event, mainly due to the neurological deficits that appear, as well as dysphagia [18]. These characteristics will determine the appearance of infections, ulcers and other complications, which will worsen the patient’s nutritional status. In these cases, it is essential that the food provided is based on texture-modified foods, with a high caloric density, as well as hyperproteic foods [19]. When this is not possible, supportive feeding by nasogastric tube can be used [20]. Additionally, in these patients we found a microbial profile similar to healthy controls in terms of α-abundance and structure; it was found that patients with LCA presented more producers of short-chain fatty acids, including *Odoribacter*, *Akkermansia*, *Ruminococcaceae*_UCG_005 and *Victivallis*. These data indicate that these patients had significant dysbiosis [21]. In addition, Hemorrhagic Transformation (HT) was found to correlate with serum levels of SCFA, especially butyrate, and with the inflammatory response [22].

In neurodegenerative diseases, a differentiated microbial profile has been repeatedly shown in comparison with control groups, with an increase in the permeability of the intestinal barrier. Specifically in AD, an increase in proinflammatory bacteria and a decrease in anti-inflammatory bacteria has been found, which in turn favors the increase in different metabolites (proinflammatory cytokines, among others) and a decrease in other metabolites, such as butyrate, with antioxidant and immunomodulatory properties [23]. On the other hand, in these patients there is a high risk of energy-protein malnutrition, so nutrition is essential in the development of the pathology when oropharyngeal dysphagia is present, as well as other physiological and autonomic alterations [24,25]. Another example is PD, where we found a dysbiosis marked by a higher abundance of Helicobacter pylori, which is associated with a worsening of the characteristic symptomatology of this disease. Studies along these lines have found a lower presence of *Prevotellaceae* and an increase in *Enteroacteriaceae*, which is related to high levels of neuroactive [26,27]. In addition, these patients had higher intestinal permeability. We know that both *Prevotellaceae* and *Enteroacteriacea* are related to motor symptomatology in PD (postural and gait difficulties [28]).

In these patients, apart from controlling nutrition for the obvious benefits of maintaining microbial homeostasis, it is also important to keep in mind that these patients are more susceptible to osteoporosis, so the diet should be focused on the inclusion of calcium and vitamin D in abundance [29]. Other studies have been carried out that focused on the nutritional and metabolomic profile in neurological pathologies such as MS and ALS, finding dysbiosis associated with the evolution of the pathology [30,31]. These results indicate a promising line of study for the approach to neurological diseases. Knowing the direct relationship between dysbiosis and these pathologies, and focusing on the molecular study of the bacterial composition of the intestine, it is possible to apply holistic interventions that allow for the improvement of the quality of life of patients and the reduction in the associated symptomatology by intervening in human nutrition [32]. Another factor that can modulate certain symptoms associated with neurological diseases is the practice of sports. Currently, the benefits of frequent Physical Activity (PA) for the physical and mental health of individuals have been demonstrated, improving physical condition and the regulation of the immune system [33,34]. In line with this, a recent review also notes the benefits of regular exercise in mood improvement, stress management, and social skill enhancement, particularly when combined with mindfulness practice [35].

Disability of neurological origin as a result of stroke, TBI or spinal cord injury is very high. In line with this, several studies have shown that sports practice has the capacity to improve the neurological level (NS) [36]. Specifically, it was shown that PA has neuroprotective functions and activates different mechanisms, such as the control of breathing and heart rate, increased blood flow or the regulation of glucose levels, among others [34]. Furthermore, PA also improves brain function through the synthesis and release of neurotransmitters during exercise [37]. In addition, PA has a protective function in neuronal proliferation and maintenance; it favors a balanced nutrient supply while maintaining glucose homeostasis; it maintains oxygen homeostasis; it intervenes in cerebral vascularization processes; it favors synaptic plasticity, among others [38,39].

Although it is always important to take into account the most appropriate type of PA for each individual, the benefits of sports practice for general health are very high and wide-ranging, regardless of factors such as age, weight, gender, etc. PA is a natural neuroprotector for all people [40]. And at the neurological level, it is possible to affirm that global cognitive function is improved, as well as executive functions; neurogenesis is stimulated and synaptic growth is increased [41,42]. The latest research data on the etiopathogenesis of neurological diseases, nutrition and sports practice are essential. The understanding of molecular and clinical mechanisms allows for a multidisciplinary approach in the intervention of these complex pathologies.

## 3. Interconnections between Physical Activity and Neurodegenerative Disorders

The knowledge about the benefits of exercise in humans is increasingly extensive. Beyond its well-known role as cardioprotective, scientific evidence has shown that exercise also enhances various brain functions, positioning it as a significant neuroprotector and therapeutic tool in the prevention of mental disorders and neurodegenerative diseases [43]. Especially in an increasingly aging population, physical exercise proves to be an effective tool in treating neurodegenerative pathology, while improving functional capacity, mobility, and autonomy in dementia patients [44]. However, it is disheartening to see that, despite the growing evidence, protocols to incorporate physical exercise into dementia treatment are not promoted [44].

Cognitive decline represents a real socioeconomic and health challenge in a society with an increasingly longer life expectancy. Pharmacological treatments have not proven to be truly effective in addressing cognitive problems, leading to a growing interest in healthy lifestyles. In this context, physical exercise stands out as a non-pharmacological treatment that promotes neuroprotection and enhances cognitive function in neurodegenerative pathology [45]. It is important to note that physical exercise, among its many advantages, lacks undesirable side effects commonly associated with pharmacological treatments, making it a non-pharmacological treatment capable of harmonizing brain function and structure [46]. Additionally, patients with neurodegenerative diseases often experience sleep disorders, which not only affect their quality of life but also pose a potential risk for accelerating disease progression. Once again, physical exercise emerges as the best solution for mitigating sleep disorders and alleviating neurodegeneration, especially in Parkinson’s and Alzheimer’s diseases [47].

In this context, the neuroprotection processes induced by physical activity are attributed to increased production of neurotransmitters and neurotrophic factors, as well as neural hormone production. Physical activity provides neuroprotection and neuroprevention by promoting better cognition, memory, sleep, angiogenesis in the nervous system, and reducing stress, anxiety, insulin resistance, and neuroinflammation [43]. Physical exercise, especially aerobic exercise, benefits synaptic plasticity, particularly in terms of learning, memory, and motor function. It is known that physical exercise delays the progression of neurodegenerative pathology by acting on the nervous system, reducing the accumulation of neurotoxic amyloid aggregates and limiting oxidative stress, neuroinflammation, and neuronal death. Indeed, skeletal muscle, in response to physical exercise, secretes various neurotrophic factors that are neuroprotective [48]. In this context, physical exercise practice is key for patients with neurodegenerative pathology because it increases hormone, neurotransmitter, and neurotrophic factor production. Additionally, the nervous system benefits from physical exercise. Its practice sensitizes the parasympathetic nervous system, the autonomic nervous system, and the central nervous system by promoting synaptic plasticity, neurogenesis, angiogenesis, and autophagy [49].

Recent discoveries show how physical activity could provide protection in pathologies such as Alzheimer’s disease, Parkinson’s, and Huntington’s by positively regulating synaptic signaling pathways. Regarding frontotemporal dementia, it is associated with the repair of mitochondrial function and energy precursors. These advances indicate that physical activity directs transcriptional changes in the brain, reconnecting different pathways against neurodegeneration [50]. In fact, in neurodegenerative disorders such as multiple sclerosis, Alzheimer’s disease, mild cognitive impairment, and Parkinson’s disease, which are related to lower levels of brain-derived neurotrophic factor, the role of physical exercise vs. no exercise has been investigated. It has been concluded that exercise interventions increase plasma levels of brain-derived neurotrophic factor in patients with neurodegenerative pathology [51]. Indeed, the preventive role of exercise is key in neurodegenerative pathology, as it influences the epigenetics of neurotrophic factor and controls epigenetic mechanisms in critical areas such as the hippocampus for memory regulation. Therefore, regulating epigenetic mechanisms through physical exercise is related to greater prevention and better prognosis in the development of neurodegenerative pathology [52].

Among the multiple benefits that physical exercise can elicit in the body, it is also related to the endocannabinoid system. Recent studies have shown that physical exercise correlates with increased serum concentrations of endocannabinoids and higher expression of the cannabinoid receptor type 1 in the brain. This results in reduced neuroinflammation, improvements in memory, antidepressant effects, and benefits in neuroplasticity. These discoveries could be helpful both preventively and therapeutically in neurodegenerative pathology [53]. Research in this field also focuses on the study of biomarkers and physical activity, presenting encouraging advances. Specifically, Raffin et al. in their 2021 study found that achieving at least 90 min of physical activity per week (excluding light physical activity) is associated with a lower likelihood of having elevated concentrations of neurofilament light chain proteins. Control of this biomarker is key in controlling neurodegenerative pathology. In fact, having elevated levels of neurofilament light chain proteins decreases the cognitive benefits of physical activity. However, physical activity could be crucial in alleviating the harmful relationship between amyloid burden and cognitive functions [54]. Future advances in the prevention and treatment of neurodegenerative pathology will continue to be linked to physical exercise. We find very promising lines of research for an increasingly aging population. As a consequence, the prevalence and incidence of neurodegenerative pathology will continue to grow.

## 4. Dietary Patterns and Their Influence on the Progression of Neurodegenerative Diseases

Recent findings demonstrate how physical activity could provide protection against pathologies such as Alzheimer’s disease, Parkinson’s disease, and Huntington’s disease by positively regulating synaptic signaling pathways. Regarding frontotemporal dementia, it is associated with the repair of mitochondrial function and energy precursors. These advances indicate that physical activity directs transcriptional changes in the brain, reconnecting different pathways against neurodegeneration [50]. In fact, in neurodegenerative disorders such as multiple sclerosis, Alzheimer’s disease, mild cognitive impairment, and Parkinson’s disease, which are related to lower levels of brain-derived neurotrophic factor, the role of physical exercise vs. no exercise has been investigated, and it has been concluded that interventions with physical exercise increase plasma levels of brain-derived neurotrophic factor in patients with neurodegenerative pathology [51]. Indeed, the preventive role of exercise is key in neurodegenerative pathology, since it influences the epigenetics of the neurotrophic factor and controls epigenetic mechanisms in fundamental areas such as the hippocampus for memory regulation. Therefore, regulating epigenetic mechanisms through physical exercise will be related to greater prevention and better prognosis in the development of neurodegenerative pathology [52].

Among the many benefits that physical exercise can bring to the body, it is also related to the endocannabinoid system. Recent studies have shown that physical exercise correlates with increased serum concentrations of endocannabinoids and increased expression of cannabinoid receptor type 1 in the brain. This translates into less neuroinflammation, improvements in memory, antidepressant effects, and benefits in neuroplasticity. These findings could be helpful both preventively and therapeutically in neurodegenerative pathology [53]. Research in this field is also focused on the study of biomarkers and physical activity, presenting encouraging advances. Specifically, Raffin et al. in their 2021 study found that achieving at least 90 min of physical activity per week (excluding light physical activity) is associated with a lower likelihood of having elevated concentrations of neurofilament light chain proteins. Controlling this biomarker is key in controlling neurodegenerative pathology. In fact, having elevated levels of neurofilament light chain proteins diminishes the cognitive benefits of physical activity. However, physical activity could be fundamental in mitigating the harmful relationship between amyloid burden and cognitive functions [54]. Future advances in the prevention and treatment of neurodegenerative pathology will continue to be linked to physical exercise. We find very promising lines of research for an increasingly aging population. Consequently, the prevalence and incidence of neurodegenerative pathology will continue to grow.

Recent research reveals how physical activity could offer protection against conditions such as Alzheimer’s, Parkinson’s, and Huntington’s diseases by positively regulating synaptic signaling pathways. In the case of frontotemporal dementia, it is associated with the restoration of mitochondrial function and energy precursors. These advancements suggest that physical activity influences transcriptional changes in the brain, reconnecting various pathways against neurodegeneration [50]. In fact, neurodegenerative disorders such as multiple sclerosis, Alzheimer’s disease, mild cognitive impairment, and Parkinson’s disease, which are linked to lower levels of brain-derived neurotrophic factor, have been studied in terms of the role of physical exercise versus no exercise. The conclusion drawn is that interventions involving physical exercise increase plasma levels of brain-derived neurotrophic factor in patients with neurodegenerative conditions [51]. Furthermore, the preventive aspect of exercise is pivotal in neurodegenerative pathology, as it influences the epigenetics of neurotrophic factors and controls epigenetic mechanisms in vital areas such as the hippocampus for memory regulation. Hence, regulating epigenetic mechanisms through physical exercise is associated with greater prevention and improved prognosis in neurodegenerative conditions [52].

In a different context, the beneficial effects of intermittent or chronic food restriction are presented. This practice positively impacts brain metabolism, intestinal microbiome biology, and immunity. The interaction between calorie intake, meal periodicity, food quality, and gut microbiome is related to specific molecular and metabolic pathways that modulate cell homeostasis at the tissue and organ levels. This, in turn, influences brain inflammation during aging and neuroinflammatory pathologies of the central nervous system. Therefore, intermittent caloric restriction, which is easier to implement, and chronic caloric restriction, which is more challenging to apply, could have preventive and therapeutic aspects concerning neurodegenerative pathology [55]. Along this line, microglia and astrocytes are glial cells involved in modulating such neuroinflammation. The pro-inflammatory response of these central nervous system glial cells suggests that it can be mitigated through caloric restriction. However, the molecular-level explanation for this phenomenon continues to elude researchers [56]. Indeed, the central nervous system is highly vulnerable to the aging process, leading to metabolic, structural, and functional alterations in the brain. These alterations result in cognitive decline. Caloric restriction reduces oxidative stress, enhances mitochondrial function, improves anti-inflammatory capacity, and aids in increasing synaptic plasticity. Thus, caloric restriction reduces neurodegeneration and enhances cognitive functions [57]. Fasting and caloric restriction provide neuroprotection by increasing levels of β-HB, FGF21, and ghrelin in Parkinson’s disease. Additionally, ketogenic and protein-restricted diets have a very positive impact on alleviating motor deterioration and fluctuations. However, the Western diet correlates with a higher incidence and severity of Parkinson’s disease due to increased neuroinflammation and undesirable modifications in the gut microbiome [58].

By 2030, the global population aged over sixty-five is projected to reach 12%, with dementia predicted to affect seventy-five million individuals by that same year. Alzheimer’s disease represents the primary form of dementia. Currently, there are no available medications that can satisfactorily cure Alzheimer’s disease. In light of this, preventive interventions aim to alleviate the burden on the aging population suffering from cognitive decline. To this end, dietary habits resembling the Mediterranean diet—characterized by high consumption of plant-based foods, fruits, vegetables, nuts, with fish and olive oil as primary fat sources—appear to slow down or prevent cognitive decline and reduce the incidence of Alzheimer’s disease [58]. Age becomes the most common risk factor for dementia onset, correlating with a decline in metabolic processes and cellular bioenergetics. Along this line, type II diabetes is a risk factor for Alzheimer’s disease. Consequently, a balanced diet such as the Mediterranean diet could mitigate the risk of type II diabetes and confer neuroprotection. However, the mechanisms underlying these diet-associated neuroprotective effects remain unclear [59].

Besides age, other risk factors contribute to cognitive function decline. Determinants such as education, genetics, or environmental, demographic, and socioeconomic factors play a role in the origin and development of neurodegenerative pathology. In this regard, nutrition emerges as a potent strategy from a public health perspective. Therefore, it is imperative to understand the synergistic effects of food combinations, as they may extend beyond the known benefits of individual intake [60]. In this context, Barbaresko et al. in their 2020 meta-analysis review found inverse associations between fish consumption and the Mediterranean diet with Alzheimer’s disease. Additionally, moderate-quality evidence indicated associations between tea consumption and dementia, as well as Parkinson’s disease [61]. However, given the needs of an increasingly aging population, further research is warranted to determine preventive and therapeutic options that nutrition can offer to address the growing prevalence of neurodegenerative diseases.

## 5. Insights from Neuroimaging on Exercise and Nutrition’s Impact

Neuroimaging studies present a non-invasive technology and are essential in the understanding of certain pathologies, as well as their progress. It is a technology that stimulates and identifies changes in the direction of the proton rotation axis that appear in the water that makes up the tissues of the organism [62,63]. Briefly, MRI uses magnets with a large magnetic field that drives the protons in the body to align with the field produced [64]. The protons are stimulated and fight against the strength of the magnetic field and when the radiofrequency field is turned off the protons align with the magnetic field and the sensors can identify the energy released. It allows for imaging of non-bony parts and soft tissues of the body, differentiating between white and gray matter [65].

Although there may be some risk in certain health conditions, it is a technology that does not emit the harmful ionizing radiation of other techniques [66]. fMRI is a type of MRI that is specifically used to image and observe brain structures, allowing for the identification of the areas that consume more oxygen (active) during the performance of certain cognitive activities and allows for the assessment of the neurological status of individuals. In addition, MRI can be used to measure the metabolic state of the brain, identifying the maturation of neurons, astrocytes and axons [67,68]. In the study of neurological pathologies, MRI makes it possible to observe patterns of brain alterations [69]. In these cases, the hippocampus, for example, is a structure of great interest, and with MRI it is possible to determine its volume, relative size and the integrity of the structure. This facilitates comparison with healthy subjects, to try to identify a common signature present in the pathology, and to differentiate from healthy controls [70].

The brain develops from dietary substances, essential fatty acids, minerals, vitamins, amino acids. Although the idea that nutrients have an impact on brain structure is recent, studies have been conducted mainly evaluating the role of vitamins and trace elements (micronutrients) in brain function [71,72]. Thus, we know that some vitamins, such as B6 and B12, are involved in the synthesis of neurotransmitters. In addition, B12 delays the onset of symptoms associated with dementia in older adults, and in adolescents it has been observed that borderline levels of this vitamin are associated with changes in cognitive functioning. In addition, some components of vitamin E are absorbed by the brain and are involved in the protection of the nerve membrane [73,74]. Additionally, we know that the intake of foods with low glycemic levels guarantees the maintenance of a low insulin index. This will favor glucose homeostasis, which improves cognitive performance. The effects of micronutrients are currently being studied, as we know that the nutritional factor plays an essential role in brain development, its functioning, and the prediction of the appearance of neurological symptomatology [75,76].

MRI studies of infants with intrauterine nutritional deficiencies showed alterations in white and gray matter, as well as in macronutrient and micronutrient levels. In addition, it was determined that a diet rich in nutrients is related to larger volumes of the caudate nucleus, as well as a higher verbal IQ [77]. It is important to note that the studies that have been conducted addressing the effects of nutrition on the brain using neuroimaging techniques have focused mainly on the age variable, studying the impact of nutrition to improve brain development or to protect against the atrophy characteristic of advanced age [78].

Examining the impact of physical exercise on the brain, early MRI studies showed a positive relationship between brain tissue density and aerobic fitness [79]. Individuals tested showed decreased gray matter density in the prefrontal, temporal and parietal cortexes. A longitudinal study along these lines by Eriksen et al. in 2011 showed that low-intensity exercise produced an increase in hippocampal volume, and this correlated with significant improvements in amnesic function, which were maintained up to twelve months later, indicating structural plasticity mediated by physical activity [80].

Other cross-sectional studies performed with low exercise control conditions indicated that it is a modulator of cortical and subcortical brain structure. Thus, increases in cortical thickness appear, which are related to improvements in memory [81]. PET studies showed that acute exercise is associated with an increase in the metabolic rate of glucose consumption in cortical areas. It has also been shown that PA has a neuroprotective effect and reduces amyloid [82]. It is clear that neuroimaging studies make it possible to observe the benefits of both nutrition and PA on the brain. These results offer the opportunity to expand knowledge on the impact of different factors on brain function, structure and morphology.

## 6. Therapeutic Roles of Exercise and Physical Activity in Neurological Rehabilitation

The growing body of evidence indicates a pivotal role for exercise and physical activity in neurological rehabilitation, serving as a comprehensive therapeutic intervention. Systematic reviews have consistently demonstrated improvements in health parameters, global cognitive ability, and functional capacity following exercise training rehabilitation programs in various neurological diseases, such as multiple sclerosis, stroke, and Parkinson’s [83,84,85,86]. The suggested mechanisms encompass the augmentation of physical activity/exercise, resulting in enhanced oxygen consumption, increased cerebral blood flow, and the stimulation of brain cell regeneration, particularly within encephalic regions associated with cognitive function [85,87,88]. Noteworthy factors contributing to these effects include neuroplasticity, neurotrophic factors, and the modulation of neurotransmitters, providing a robust foundation for understanding the therapeutic potential.

The existing literature strongly supports the notion that physical exercise exerts positive effects on cognition, promoting neuroplasticity and serving as a preventive measure against diseases associated with cognitive decline [89]. Furthermore, exercise positively influences Brain-Derived Neurotrophic Factor (BDNF) levels in neurological populations, enhancing neuroplasticity and facilitating improved motor performance [90]. Additionally, exercise impacts neurotransmitters, neuromodulators, cytokines, and neurotrophins, thereby fostering beneficial effects on neurological disorders [91]. A previous systematic review of a systematic review indicated that exercise interventions effectively reduced the number, frequency, and rate of falls among individuals with neurological disorders, encompassing cognitive impairment, dementia, and Parkinson’s disease [86]. In the mentioned umbrella review, the predominant exercise programs implemented in neurological disorders included aerobic and resistance training. However, evidence suggests that these modalities impact distinct neuro-cognitive networks, eliciting various beneficial effects. [89].

Specifically, aerobic exercise has been employed as a therapeutic intervention in certain neurological disorders, demonstrating substantial improvements in both neurological and overall health. For example, a previous systematic review conducted on post-stroke patients affirms that aerobic exercise leads to a noteworthy enhancement in global cognitive ability and positive outcomes in specific cognitive domains, particularly memory, attention, and visuospatial ability [85]. Additionally, a recent meta-analysis in multiple sclerosis patients illustrated that aerobic exercise improves gait speed, walking endurance, balance, cardiorespiratory fitness, and perception of fatigue [83]. Intriguingly, the effect on gait speed was more pronounced when aerobic exercise is performed at low-to-moderate intensity, 3 days per week, continuous, and walking. Furthermore, aerobic exercise has shown promising results in other neurological disorders, such as dementia [92], Parkinson’s [93], or traumatic brain injury [94].

In the context of resistance training, it also induces positive effects on neurological and overall health in patients with neurological disorders. A systematic review with meta-analyses demonstrated the effectiveness of resistance training in individuals with multiple sclerosis, enhancing lower limb isometric strength and functional capacity [84]. Notably, the study suggests that employing long durations (more than 6 weeks), high intensity (more than 80% 1-RM), and a training frequency of two days per week may serve as an effective stimulus to improve strength, functional capacity, balance, and alleviate fatigue in this neurological pathology (i.e., multiple sclerosis). Significantly, the same research group suggests employing fast-velocity concentric resistance training in neurological populations. This form of resistance training involves maximum-velocity contractions during the concentric phase, resulting in heightened neuromuscular adaptations and greater improvements in functional capacity and balance compared to other types of physical training programs [95,96]. The intention to generate force at maximal velocity during the concentric phase imposes greater neural demands, indicating promising results improving health and functional markers in neurological disorders such as individuals with multiple sclerosis or Parkinson’s [97]. Overall, resistance training has proven its effectiveness as a therapeutic tool in numerous neurological disorders such as Parkinson’s [98], Stroke [99], Alzheimer [100], or traumatic brain injury [101].

However, there are limitations and challenges regarding the effectiveness of exercise in neurological disorders. Navigating the landscape of sports-based interventions in neurological rehabilitation poses inherent challenges that require careful consideration. The significant variability among individuals, both in terms of their conditions and responses to interventions, presents a complex hurdle. Accessibility issues, including factors such as facility availability and financial constraints, further contribute to the intricacy of implementation. Additionally, ensuring consistent adherence to prescribed exercise regimens proves to be a notable challenge, demanding commitment from individuals who may already contend with neurological conditions. Concerning adherence, the use of exercise-based games, including virtual reality and interactive video game interventions, has emerged as an intriguing research avenue in recent years. This approach holds promise due to increased motivation and enjoyment for patients, potentially leading to greater treatment adherence and demonstrating at least comparable effectiveness to traditional therapy in individuals with neurological diseases [102].

Concerning the clinical implications and future research, it is crucial to bridge the gap between research insights and actionable strategies for healthcare professionals. The practical implications highlight the significance of incorporating evidence-based sports interventions into clinical settings, thereby enhancing neurological rehabilitation practices. Moving forward, the focus shifts towards future research directions, emphasizing the necessity for tailored interventions that consider individual variability and optimize protocols for maximal efficacy. Additionally, there is a clear imperative to address specific neurological conditions with targeted approaches, ensuring a nuanced understanding of diverse disorders and tailoring interventions accordingly. This dual perspective, aimed at translating research findings into clinical application and guiding future investigations, serves as a pivotal avenue to advance the field of neurological rehabilitation.

## 7. Dietary Interventions for Neuroplasticity Enhancement and Recovery in Neurological Rehabilitation

In recent years, there has been a growing interest in exploring the role of nutrition as a key factor that may promote neuroplasticity and facilitate recovery in neurological disorders. Neuroplasticity is related to the fundamental principles of brain adaptability. It could be linked to the ability of the brain to reorganize and adjust its connections and functions as a consequence of learning and experiences, even after experiencing injury. This adaptive mechanism includes numerous processes, particularly synaptic plasticity, characterized by enduring alterations in the strength of synaptic connections, and neurogenesis, the generation of new neurons. Hence, this process is believed to occur predominantly in the hippocampus [103]. Moreover, recent literature proposed how different nutrient intake levels may promote neurogenesis, synaptic plasticity, and neuronal survival. This is the case for nutritional patterns, such as Mediterranean diet, based on a diverse range of fruits, vegetables, legumes, nuts, and whole grains, which includes bioactive components such as antioxidant substances such as vitamins and polyphenols, other phytochemicals, such as curcumin, and unsaturated fatty acids [104].

Firstly, omega-3 fatty acids have been described by recent literature as important contributors to the health of brain maintenance [105]. Hence, docosahexaenoic acid (DHA) and eicosapentaenoic acid (EPA) are essential components of cell membranes in the brain. These fatty acids contribute to the fluidity and integrity of cell membranes, influencing signal transduction and synaptic plasticity [106,107]. In line with this, several studies have suggested that a diet rich in omega-3 fatty acids may enhance neuroplasticity and contribute to improved cognitive function. This importance is associated with their anti-inflammatory and antioxidant effects, which serve to safeguard neurons [108,109]. Moreover, recent authors found a significant association between omega-3 fatty acid consumption and the reduction in, or improvement of, different cognitive disorders, such as Parkinson’s disease, Alzheimer’s disease and the recovery of stroke and traumatic brain injury [110,111,112,113]. For example, regarding Parkinson’s disease, it has been pointed out by prospective studies how consuming omega-3 polyunsaturated fatty acids (PUFAs) was significantly linked to a reduced risk of Parkinson’s disease [114]. Moreover, a six-month treatment involving a daily intake of 800 mg of DHA and 290 mg of EPA from fish oil revealed a 50% reduction in those patients treated with DHA in the total score of the Hamilton Rating Scale for Depression (HDRS) compared to the placebo group, which utilized corn oil. The DHA intake triggered a significant improvement in depressive symptoms associated with Parkinson’s disease [115]. These results were consistent with recent research, as in a randomized double-blind placebo-controlled clinical trial involving 60 Parkinson’s disease patients, the administration of 1000 mg of omega-3 fatty acids from flaxseed oil added to 400 IU of vitamin E supplements over a three-month period led to an improvement in unified Parkinson’s disease rating scale (UPRDS), a stratification scale used to monitor the progression of Parkinson’s disease [116]. Finally, concerning traumatic brain injury, it has been proposed by recent literature how the consumption of a combination of omega-3 polyunsaturated fatty acids (DHA and EPA) and vitamin D3 over a period of one month significantly reduced to pre-injury plasma levels of three of most important biomarkers known as T-tau, GFAP, and UCH-L1 [111], which has been strongly associated with the presence of neurotrauma [117]. These outcomes could be explained by the omega-3 fatty acid effects at a molecular level, since they may have a positive impact on cellular elements such as proteins, receptors, ion channels, and enzymes, triggering a decrease in inflammation levels, improving synaptic plasticity and dendritic spines, as well as enhancing neurotransmission and signaling pathways. Additionally, they increase Brain-Derived Neurotrophic Factor (BDNF) and synaptic protection, constituting synaptic protection [118,119,120,121,122,123].

Secondly, some antioxidant substances, such as vitamin C, also has been described by their potential effect of enhancing neuroplasticity and promoting neural recovery. In line with this, vitamin C (ascorbic acid) has been pointed out by its primarily function as an antioxidant, scavenging oxygen species, and acting as an enzymatic cofactor, suggesting its potential activity as a neuroprotective agent in several diseases, such as neurodegenerative diseases, ischemia, stroke, and traumatic brain injury, as evidenced by old and recent preclinical and clinical investigations [124,125]. However, it has also been associated with a direct key activity as a neuromodulator due to its capability regulating neurotransmitters, GABA and NMDA receptors, and calcium channels [126,127,128,129], and it has been highlighted by its ability-regulating signaling pathways linked to both cell survival and synaptic plasticity [130]. Moreover, a lack of ascorbic acid has been linked to deficits in spatial memory, diminished hippocampal volume, and decreased neuronal populations in the dentate gyrus of the hippocampus [131]. In mouse models, it has been described how diminished ascorbic acid levels in neuroblastoma cells not only compromise dendritic morphology, maturation, and complexity but also point out the potential positive influence of ascorbic acid on synaptic plasticity, dendritic development, and neural function [132]. Moreover, the important role that vitamin C may play in Alzheimer’s disease has been highlighted by recent literature, as it has been described in mouse models how a 6-month treatment with ascorbic acid not only reversed behavioral deficits but also reduced the formation of amyloid-β (A-β) oligomers. Additionally, this reduction in amyloid-β oligomerization was accompanied by a decrease in brain oxidative damage and a lowered ratio of soluble Aβ42 to Aβ40, a key indicator of disease progression. Furthermore, the study demonstrated that ascorbic acid reinstated the diminished levels of brain synaptophysin and the phosphorylation of Tau at Ser39 [133]. All these findings may be explained since ascorbic acid plays a critical role in neuromodulation, as it has been related to increased BDNF levels, as recent literature pointed out [134,135], promoting neuroplasticity.

Regarding other antioxidant substances, such as polyphenols, it has been largely described how the Mediterranean diet is composed of different types of flavonoids extracted from grains, vegetables, fruits, and some drinks such as red wine, tea and coffee [136]. In line with this, these substances have been extensively related to the prevention of different pathologies, due to its antioxidant activity. Regarding Alzheimer’s disease, polyphenols present in raspberries and blueberries protect against Aβ neurotoxicity. This fact may be explained due to polyphenol’s capability of inhibiting Aβ oligomerization, as it may increase Aβ42 monomer clearance. This activity is subsequent to monomer modulation in order to modify interactions as well as to oligomer’s remodulation into nontoxic forms [137,138,139,140]. Moreover, recent literature proposed how polyphenols may modulate Tau hyperphosphorylation as well, as they could be responsible for the reduction in Tau B-sheet production [141].

Finally, it has been extensively pointed out by the latest literature how vitamin E, (α-tocopherol) offers useful protection against lipid peroxidation, DNA mutations, mitochondrial damage, loss of neurons and Aβ accumulation. Moreover, the same researchers proposed how raised γ-tocopherol levels were related to lower Aβ accumulation, as well as how they were linked to fewer neurofibrillary tangle t-pathology. This fact could be explained since researchers proposed how the consumption of vitamin E supplements, which were only composed of α-tocopherol, triggered a rapid reduction in γ-tocopherol levels, which consequently could be related to increased Alzheimer pathology [142]. Additionally, recent literature described how the use of α-tocopherol combined to γ-tocopherol may be linked to the postponement of age-related cognitive decline and a reduced risk of Alzheimer’s disease [143].

## 8. Advancements in Technology for Evaluating the Impact of Sports and Nutrition on Neurological Health

The relationship between sports, nutrition, and neurology has won significant attention in recent years. Understanding how physical activity and dietary habits influence neurological health is crucial for optimizing performance, preventing neurodegenerative diseases, and enhancing overall well-being. Advances in technology have revolutionized the assessment of these effects, offering researchers powerful tools to explore this intricate relationship.

In line with this, recent literature proposed the use of different techniques to improve the knowledge of sports and nutrition effects on neurology, firstly proposing the use of wearable devices. Thus, wearable sensors, such as fitness trackers and smartwatches, have become popular in sports and exercise settings. These devices can monitor various physiological parameters, including heart rate, sleep patterns, and physical activity levels. By analyzing this data, researchers can gain insights into the effects of different sports activities on neurological function. For example, in a study conducted recently, researchers aimed to evaluate the validity of six frequently utilized wearable devices, including Apple Watch S6, Garmin Forerunner 245 Music, Polar Vantage V, Oura Ring Generation 2, WHOOP 3.0, and Somfit, in measuring sleep parameters. Additionally, they aimed to assess the accuracy of these same devices in monitoring heart rate and heart rate variability either during nighttime sleep or in the period just before it [144]. Findings in this research suggest that all six devices demonstrate validity in accurately assessing the timing and duration of sleep in field-based settings. However, analyses utilizing the multi-state classification of sleep reveal that improvements are needed for all six devices to accurately assess specific sleep stages.

Regarding HRV (Heart Rate Variability) use, recent research proposed its utility as a useful tool in the evaluation of the physiological state of an organism which could be assessed through autonomic modulation. Hence, analysis of HRV in a sample of male patients which presented mixed anxiety and depression disorder revealed that they showed sympathetic hyperarousal at the onset of the research, characterized by insignificant PNN50 values and a predominance of low-frequency activity, as sympathetic hyperactivation experienced a slight decrease after the intervention proposed in the present research [145]. Concerning epilepsy, HRV has been largely studied in last three decades, and it has been demonstrated to be helpful in epilepsy management. In line with this, recent authors proposed how HRV recorded in the period between seizures indicated a modification in autonomic balance towards sympathetic dominance, which tends to lead to increased sympathetic activity [146]. Furthermore, in a recent study that involved 238 temporal lobe seizures from 41 patients, HRV parameters showed significant modifications, such as reduced RR and pNN50, which aided to recognize the pre-ictal state in 90% of patients and 41% of seizures [147,148]. Finally, HRV has also shown beneficial properties in the early detection and differentiation of several diseases, as in a retrospective analysis of resting HRV among patients with mild cognitive impairment. Those who later developed dementia with Lewy bodies presented lower levels of HRV (including SDNN, RMSSD, LF, HF) compared to those who progressed to Alzheimer’s disease [149].

In terms of neuroimaging technologies, functional Magnetic Resonance Imaging (fMRI) and electroencephalography (EEG) have shown beneficial properties that allow researchers to observe brain activity in response to sports participation and nutritional interventions. These techniques provide valuable information about changes in neural connectivity, neurotransmitter levels, and brain structure associated with physical exercise and dietary modifications [150]. In line with this, a study of 165 cognitively healthy older adults aged between 59 to 81 years conducted a maximal graded exercise test to assess their cardiorespiratory fitness. Additionally, their hippocampal volume was measured using functional Magnetic Resonance Imaging (fMRI) while they performed a spatial memory task. The results showed a positive correlation between higher fitness levels and larger bilateral hippocampal volume. Furthermore, increased fitness levels and larger hippocampal volume were linked to better performance in spatial memory tasks [151,152]. Moreover, the same researchers demonstrated later how, in a study of 120 adults, the exercise treatment group showed a significant 2% increase in their hippocampal volume. Furthermore, this group presented elevated levels of serum BDNF related to enhancements in spatial memory abilities. These findings conclusively support the notion that aerobic exercise training contributes to an increase in hippocampal volume during later stages of life, thereby positively impacting memory performance [80]. Regarding EEG, previous studies investigated a specific type of EEG activity called event-related brain potentials (ERPs). Thus, ERPs have been identified as especially responsive to physical activity and cardiorespiratory fitness levels. These ERPs represent a category of EEG activity triggered either by a stimulus or in anticipation of a response [153]. Moreover, a notable ERP component was observed after committing an error known as Error-Related Negativity (ERN). Findings in recent literature revealed that older adults experienced more pronounced reaction time delays in task conditions demanding higher levels of cognitive control, which added to smaller ERN amplitudes, compared to younger adults. Additionally, when physical activity levels were considered, both older and younger physically active participants exhibited reduced ERN amplitudes and greater post-error response delays compared to their sedentary corresponding controls [154]. Hence, according to these results, it could be considered that neuroimaging technologies such a fMRI and EEG have demonstrated the positive effects of exercise on cognitive function and brain plasticity.

Regarding nutrition aspects, metabolomics is a revolutionary approach that involves the comprehensive analysis of small molecules (metabolites) present in biological samples, such as blood or urine. By employing techniques such as mass spectrometry and nuclear magnetic resonance spectroscopy, researchers may identify metabolic signatures associated with specific dietary patterns and nutritional interventions. Then, it allows for a precise assessment of how dietary factors influence neurochemistry and neurological health. For example, recent literature proposed how differences in metabolomic marks were found in patients with major depression. In line with this, the three symptom dimensions presented different metabolites in those patients. Thus, the severity of the immunometabolic dimension, characterized by unusual and energy-associated symptoms such as raised appetite, weight gain, excessive sleepiness, decreased energy levels, and a sensation of leaden paralysis, was primarily associated with gut-derived metabolites, as well as with a selection of acylcarnitines and long-chain saturated free fatty acids. Conversely, the severity of the melancholia dimension showed significant correlations with various phosphatidylcholines, particularly those of the ether-linked variety, lysophosphatidylcholines, as well as certain amino acids. Additionally, the severity of anxious distress presented significant correlations with numerous medium and long-chain free fatty acids, both saturated and polyunsaturated, sphingomyelins, several amino acids, and bile acids [155].

To summarize, emerging technologies play a crucial role in advancing the understanding of the effects of sports and nutrition on neurology. Wearable devices enable real-time monitoring of physiological parameters during physical activity and sleep, while neuroimaging techniques provide insights into brain function and structure. Lastly, metabolomics offers novel approaches that may assess the impact of dietary effects on neurological health at the molecular level. By using these technological innovations, researchers may develop more effective strategies for promoting brain health and optimizing performance through sports and nutrition.

## 9. Clinical Trials Assessing Exercise Efficacy in Neurological Disorder Management

Multiple studies have shown evidence that engaging in physical activity has a substantial impact on reducing both mortality and morbidity, enhancing community involvement, and enhancing the overall quality of life in terms of health [156]. These advantages demonstrate that including higher levels of physical activity and exercise in the regular treatment of neurological illnesses is essential. For these patients, it is important to design physical activity programs that are tailored to their specific needs. This involves creating suitable environmental circumstances and ensuring safety measures, considering the evaluation of their functional state and the severity of their disease. It is important to tailor the duration, intensity, and type of planned physical exercise to everyone. Adequate rest periods should be provided during the activity, and criteria for stopping should be established based on individual tolerance [156]. For instance, individuals suffering from Chronic Neurological Disorders (CNCs) frequently exhibit imbalances in physical abilities, resulting in noticeable disparities between opposite limbs in terms of bodily activities [157]. These imbalances have been linked to decreased mobility and stability and are frequently addressed for improvement during rehabilitation. Exercise training has been proven to provide advantages for those with Chronic Neurological Conditions (CNCs), and it may also yield favorable results in terms of asymmetrical outcomes [157]. Moreover, the prevalence of chronic diseases escalates considerably as individuals grow older. Several elderly individuals are admitted to care institutions due to delirium, functional impairment, deconditioning, gait abnormalities, and falls. Hence, falls frequently indicate an inherent weakness or ailment, especially in those with neurological conditions. Older persons with neurological illnesses, such as dementia, Parkinson’s disease (PD), multiple sclerosis (MS), and stroke, have a higher incidence of falls compared to healthy older adults [86]. Regarding mental health, Adamson and colleagues’ review also presents empirical evidence that exercise, when compared to a control condition, can effectively diminish depressed symptoms across various neurologic illnesses [158].

Older persons who engage in multidomain training, which combines physical and cognitive activities, have been found to experience enhancements in both their physical and cognitive well-being [159]. For instance, the objective of the multisite StayFitLonger study is to evaluate the efficacy of a home-based computerized training program that integrates physical exercises, cognitive activities, and virtual coaching [160]. The successful outcomes of the StayFitLonger project will facilitate the progress and commercialization of a scientifically based and empirically proven application that enhances both physical and cognitive well-being, enabling individuals to live independently at home [160]. In this regard, the cognitive training intervention is efficacious and has the potential to mitigate the decline of cognitive function in patients with Mild Cognitive Impairment (MCI) [161]. However, physical activity matters. Engaging in both physical and cognitive activities has a positive impact on the cognitive and physical abilities of older persons with mild cognitive impairment, either by enhancing or preserving their performance [162].

Regarding Parkinson’s Disease (PD), it is the second most prevalent neurodegenerative disorder that impacts the elderly population [163]. The neurological impairments in Parkinson’s disease impact the musculoskeletal and balance systems, resulting in reduced mobility, postural stability, and walking ability. The clinical manifestations and psychosocial consequences of Parkinson’s disease frequently restrict the engagement of individuals with Parkinson’s disease in social and physical pursuits, thus exacerbating the deterioration of their functional capacity. However, empirical studies demonstrate that it is unwarranted to abandon hope, as the majority of them indicate that exercise can yield positive outcomes [164]. Yuan et al. found that a 6-week, hospital-based exercise program called interactive videogame based (IVGB) improved the balance, postural stability, and confidence in preventing falls in older individuals with Hoehn and Yahr (HY) stage 1–3 Parkinson’s disease (PD). Therefore, exercise training using IVGB may be used as a rehabilitation program to improve physical symptoms in older persons who have mild-to-moderate Parkinson’s disease [165]. However, intense aerobic exercise may reduce the symptoms of Parkinson’s disease, but there is a lack of reliable evidence to support this claim. Furthermore, maintaining long-term adherence continues to be difficult. Nevertheless, studies have shown that individuals with Parkinson’s disease who have modest symptoms can engage in aerobic exercise inside the comfort of their own homes [166]. This form of exercise has been found to reduce the severity of motor symptoms seen during off-states. Subsequent research should ascertain the enduring efficacy and potential disease-altering impacts [166]. Nevertheless, the intervention conducted by Ashburn and colleagues’ group did not provide any positive outcomes in a personalized, gradual, home-based program aimed at preventing falls through training in balance and strength exercises [167]. Moreover, Brandín de la Cruz et al. showed that there is viability in integrating an antigravity treadmill with an immersive virtual reality system for the rehabilitation of individuals with PD [168].

Concerning other neurological disorders, multiple sclerosis is a chronic disease that primarily affects the immune system and the central nervous system [169]. It is a leading cause of neurological disability in young adults worldwide [169]. Molhemi and colleagues have recently demonstrated that both the Virtual Reality (VR)-based and conventional balance workouts have shown efficacy in enhancing balance and mobility in individuals with Multiple Sclerosis (MS) [170]. Moreover, concurrent training has influenced this type of patient and has proven to be very beneficial. There is a study that examines the impact of a 12-week intervention that combines resistance and aerobic exercise on various factors, including balance, walking ability, fatigue perception, quality of life, and disease severity, in people diagnosed with MS [171]. The patients exhibited a high level of tolerance towards the combined training, which resulted in a notable enhancement in their quality of life. This improvement was evident in their increased walking and balance capabilities, as well as a reduction in symptoms of depression, weariness, and disease severity. The findings of this study validate the positive impacts of physical activity in individuals with MS and endorse the utilization of a blend of resistance and aerobic exercise training to attain functional and psychological therapeutic results [171]. Moreover, hydrotherapy is a legitimate kind of rehabilitation for individuals diagnosed with MS. However, it is advisable to combine this scientific approach with traditional physical treatment. However, further research involving a greater sample size and comprehensive follow-up over different time periods is necessary to validate the existing findings [172].

Additionally, statistical data indicate that during the early 21st century, more than 35 million individuals were affected by Alzheimer’s Disease (AD), a progressive neurological ailment that is among the leading causes of dementia. According to the prognostic estimations of Alzheimer’s Disease International (ADI), the number of patients suffering from Alzheimer’s disease is expected to climb twofold in the next 20 years. This might potentially result in a serious social crisis, necessitating the implementation of a comprehensive dementia plan to effectively handle this substantial rise [173]. A recent study aimed to examine the distinct impacts of aerobic exercise training (AT), resistance exercise training (RT), and combination exercise training (CT) on cognitive function in older persons who reported Subjective Memory Complaints (SMCs). This study conducted by Makino et al. indicates that AT intervention has the potential to enhance delayed memory in older adults living in the community, especially in those who do not exhibit any objective memory deterioration [174]. Lewy Body Dementia (LBD) is a very aggressive form of dementia characterized by a rapidly changing disease course, a greater likelihood of negative occurrences, and a lower level of functional independence compared to Alzheimer’s disease dementia. Non-pharmacological interventions, such as progressive, high-intensity exercise, have shown effectiveness in treating other neurological groups, but their evaluation in LBD has been limited. In this regard, the PRIDE study is the initial trial that focuses on persons diagnosed with LBD. It offers significant knowledge for the development of bigger, randomized studies to further assess the effectiveness of progressive, high-intensity exercise as a viable treatment for LBD [175]. Concretely, significant enhancements were also observed in functional independence, cognition, physical function, and strength [175] (Table 1).

In summary, optimal research suggests that it is advisable to enhance levels of physical exercise in individuals with neurological disorders. Exercise and physiotherapy programs appear to be the most effective techniques to accomplish this objective [176]. Due to the diversity in the nature and prescription of exercise, it is a requirement to promote more concise protocols that can be easily disseminated among patients.

## 10. Role of Specific Nutrients in Neurological Function and Recovery

**Figure 1:**
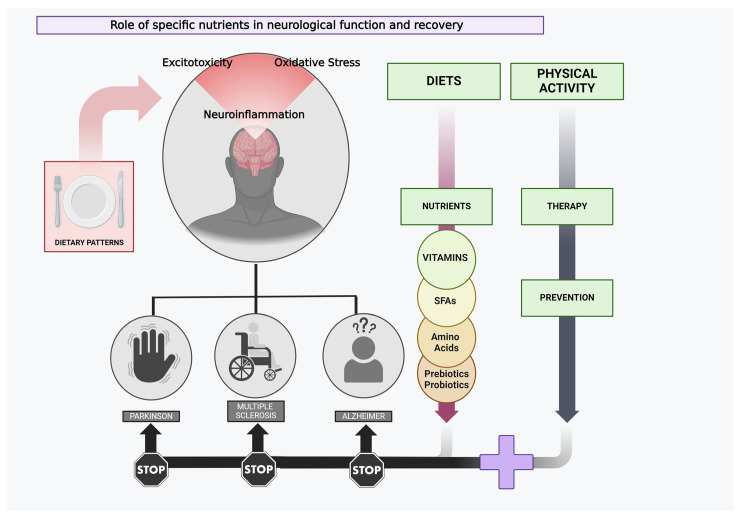
Nutritional modifications that provide high-quality nutrients that alleviate cognitive symptoms can help reduce the negative consequences of dietary choices that impede or create cognitive issues.

There has been a longstanding suspicion that the varying levels of certain nutrients can impact cognitive functions and emotions [177]. Recent research has uncovered additional insights into how dietary variables impact neuronal function and synaptic plasticity, shedding light on the crucial mechanisms behind the effects of nutrition on brain health and mental performance (Figure 1) [178]. Multiple gastrointestinal hormones with the ability to penetrate the brain, or that are synthesized inside the brain, have an impact on cognitive function. Furthermore, established regulators of the ability of synapses to change and adapt, such as brain-derived neurotrophic factor, can also work as metabolic regulators, reacting to signals from the body’s periphery, such as food consumption [178]. In fact, research indicates that the Gut Microbiota (GM) has the potential to impact the well-being of the host. Various factors, including food, medication usage, lifestyle choices, and geographical location, might alter the composition of the GM. The imbalance of gut microorganisms can disrupt the immunological balance in the brain by means of the communication pathway between the microbiota, gut, and brain. This disruption can have a significant impact on the development of neurodegenerative illnesses such as dementia and Alzheimer’s disease [179]. Comprehending the molecular foundation of the impact of food on cognition will enable us to ascertain the most effective methods of manipulating diet to enhance the resilience of neurons against harm and enhance mental acuity [180].

Moreover, neurological illnesses can alter the nutritional status of patients by directly or indirectly impacting their dietary intake. This can occur through various processes, including dysphagia (difficulty swallowing), mobility abnormalities, cognitive impairment, and depression [181]. Malnutrition exacerbates problems, leading to a delay in rehabilitation and an increase in morbidity and mortality rates. Preventing malnutrition in individuals with neurological illnesses and improving their nutritional status is crucial. This can be achieved by promptly diagnosing any nutritional decline and applying suitable nutritional therapies [182]. Conversely, as mentioned, diets can also be a contributing factor to the development of certain illnesses. Below, we detail the types of diets that can provide benefits and the specific nutrients that can enhance health.

### 10.1. Type of Diets

Various nutrition-based interventions have been suggested to improve cognitive function. These therapies frequently involve the ingestion of nutritional supplements and adherence to Dietary Restrictions (DR). First, the method involved the ingestion of particular nutrients derived from diverse sources, including plant-based extracts, minerals, vitamins, amino acids, metals, fibers, prebiotics, and saturated fats. However, the second procedure involves limiting specific foods and vitamins, such as dietary carbohydrates and amino acid composition, or adopting a time-restricted dietary regimen, such as intermittent fasting or a fasting-mimicking diet [183]. For instance, dementia risk was found to be higher in individuals with higher intake of total sugars and carbohydrates [184]. The risk of dementia was also higher in individuals with the highest or lowest fat intake compared to those with moderate intake. Lastly, individuals with the highest protein intake had a greater risk of dementia compared to those with moderate intake [185].

Regarding types of diets, research conducted on an animal model of Alzheimer’s disease suggests that the Ketogenic Diet (KD) may have a positive impact on this medical condition. This diet is characterized by a high fat content, low carbohydrate intake, and regular protein ingestion. The KD was discovered to decrease the quantities of resolved amyloid-beta in homogenates of mouse brains [186]. Furthermore, it was noted that the prolonged administration of ketone body esters to mice resulted in enhanced cognitive abilities and a decrease in beta-amyloid and highly phosphorylated tau proteins in the brain [187]. According to Reger et al. [187], the oral intake of Medium-Chain Triglycerides (MCT) resulted in an increase in ketone body levels in the blood, which could potentially enhance cognitive performance in elderly individuals with memory impairments. Moreover a KD demonstrated notable enhancements in both motor and nonmotor symptoms among individuals with PD [188]. Regarding the Mediterranean Diet (MD), it is a dietary pattern that stands out due to its food variety, low calorie content, and high intake of fruits, vegetables, legumes, nuts and seeds, grains, and fish. In contrast, it involves relatively lower consumption of meat and dairy products. For instance, a meta-analysis conducted by Papadaki et al. [189] discovered that consistent utilization of the Mediterranean Diet (MD) decreased the likelihood of experiencing a stroke. Additionally, it was observed that polyphenols derived from fruits and vegetables have the ability to regulate tau hyperphosphorylation and beta amyloid aggregation in animal models of Alzheimer’s disease [136]. Adopting a specific nutritional diet or therapy is likely done to affect physiological processes or metabolic pathways, potentially providing therapeutic advantages. These approaches can be employed to maintain a condition of dietary balance in order to mitigate the advancement of symptoms in neurological illnesses in initial research and/or medical contexts [190].

### 10.2. Nutritional Supplements Focused on Recovery

The primary shared factors that contribute to the development and advancement of neurodegenerative illnesses are oxidative stress, neuroinflammation, aging, elevated homocysteine levels, and a persistent decline in certain micronutrients in the blood plasma. Insufficient quantities of micronutrients diminish the functioning of antioxidant enzymes, potentially resulting in the oxidation and crosslinking of DNA, proteins, and fatty acids [191]. This can also lead to a depletion of mitochondrial ATP, thereby playing a role In the development of neurodegenerative illnesses [192]. Existing literature proposes that in order to effectively tackle oxidative stress as a factor in neurodegeneration, it is necessary to concurrently address excitotoxicity [193]. However, dietary micronutrients may present a more optimal solution by mitigating all three components of the neurotoxic triad (the neurotoxic trio is defined by the presence of excitotoxicity, oxidative stress, and neuroinflammation) (Figure 1). Vitamin C, vitamin E, vitamin D, and riboflavin are essential dietary antioxidants that provide simultaneous protection against excitotoxicity, oxidative stress, and neuroinflammation [194,195]. Likewise, glutathione seems to have a direct impact on all three components of the neurotoxic triad [196]. Future dietary research should investigate the potential protective effects of increasing consumption of these micronutrients, as well as other nutrients such as vitamins B6 and B12, and magnesium, against excitotoxicity, oxidative stress, and neuroinflammation [197].

Focusing on the pathophysiology of PD is closely associated with specific cellular mechanisms, such as oxidative stress, neuro-inflammation, apoptosis, and mitochondrial dysfunction. Consequently, numerous clinical and pre-clinical investigations have documented the beneficial impact of specific dietary micronutrients on PD [198]. In fact, research has firmly proven that also administering individual polyphenols may show preventive effectiveness against several symptoms of PD. Pacifici et al.’s study shows that A5^+^, a combination of polyphenols and micronutrients, can effectively counteract the harmful processes associated with this disease. It achieves this by reducing the release of pro-inflammatory cytokines, inhibiting apoptosis mechanisms, reducing oxidative stress, and promoting the differentiation of dopamine-secreting neuronal cells. These findings indicate that A5^+^ could potentially serve as an innovative therapeutic approach for treating Parkinson’s disease and its associated problems [199].

Prior research has primarily concentrated on the overall amount of vitamin E and has indicated a beneficial impact in safeguarding against PD [200]. Nevertheless, the consumption of vitamin E through diet showed no significant variation between those with Parkinson’s disease and the control group in a recent study carried out by Alizadeh et al. [201]. There was no association found between the consumption of vitamin E through diet and the likelihood or severity of PD. However, individuals diagnosed with Parkinson’s disease exhibited a greater consumption of α-tocopherol. There was a clear correlation with PD. Additionally, α-tocopherol had a greater ability to predict the risk of PD compared to other vitamins [201]. Moreover, Alizadeh’s study found a negative correlation between the severity of PD and its motor symptoms, and the intake of β-carotene, vitamin C, riboflavin, vitamin B6, and biotin. The relationship between carotenoids, B vitamins, and the development of PD is still a subject of debate and disagreement among researchers. Consistent with that research, Kim et al. conducted a study including 104 individuals with Parkinson’s Disease (PD) and found that α- and β-carotenes were negatively correlated with the UPDRS motor score [202]. In their work on rats, Jamali et al. showed that β-carotene has a therapeutic impact on diseases related to Parkinson’s disease and prevents the death of dopaminergic cells in the substantia nigra [203]. Furthermore, Wu et al. conducted a meta-analysis investigation and determined that the consumption of dietary β-carotene, rather than vitamin A intake, may provide a safeguarding effect against PD [204]. Nevertheless, Hughes et al. conducted a meticulous investigation involving 1036 individuals with PD and found no substantial correlation between the consumption of carotenoids through food and the likelihood of developing PD [205].

Regarding dementia, the results of this Gil Martínez et al.’s comprehensive analysis indicates that the addition of B Complex vitamins, particularly folic acid, may have a beneficial impact on postponing and reducing the likelihood of cognitive deterioration. Individually, ascorbic acid and a high dosage of vitamin E demonstrated favorable impacts on cognitive performance. However, there are insufficient data to substantiate their usage. The findings from trials investigating the effects of vitamin D supplementation on cognition are inconclusive in determining the potential advantages that vitamin D may provide [206]. Nevertheless, Sachs et al. demonstrated that the administration of multivitamin-mineral treatment and cocoa supplementation may enhance cognitive resilience, thereby mitigating the progression to Mild Cognitive Impairment (MCI), although it does not have a substantial impact on reducing the occurrence of MCI during a span of 3 years [207]. However, mounting data indicate that omega-3 fatty acids (ω-3fAs), carotenoids, and vitamin E have the potential to enhance cognitive performance [208].

Moreover, prebiotics and probiotics are substances that are selectively fermented by specific bacteria, such as bifidobacteria and lactic acid bacteria, and contribute to the overall health and well-being of the host [209]. For instance, the prebiotic chitosan oligosaccharide has shown the ability to reduce memory loss in a rodent model of Alzheimer’s disease. Similarly, oligosaccharides obtained from human milk have been discovered to minimize neurological damage in the brain [210]. Moreover, multiple pieces of evidence indicate that adequate intake of essential amino acids, including tryptophan, serine, and methionine, is vital for maintaining the health of the central nervous system [211]. A recent study conducted by Porter et al. revealed that patients with AD experience a decline in memory when exposed to acute tryptophan depletion followed by the consumption of a tryptophan-free amino acid beverage, compared to those who received a placebo [212]. It is important to also note that a study has found evidence that certain Saturated Fatty Acids (SFAs), specifically palmitic acids, lauric acids, and Stearic Acid (SA), can cause the release of inflammation-related substances, such as Tumor Necrosis Factor-alpha (TNF) and interleukin-6 (IL-6), by activating Toll-Like Receptor 4 (TLR4) signaling in astrocytes grown in a laboratory setting. The findings indicate that increased levels of Saturated Fatty Acids (SFAs) have the potential to trigger the formation of tumors and inflammation in the brain, leading to harmful neurological consequences [213] (Table 2).

Thus, findings indicate once more that dietary nutrients may be a viable therapy choice for addressing brain problems. However, also it has been proposed that manipulating food intake can impact the metabolic process of the epigenetics–immunity cycle, leading to improved brain health [214]. Recent research provides a mechanistic explanation for this phenomenon [215]. It is worth mentioning that while the autonomic nervous system regulates metabolic processes, epigenetics, and immune-mediated responses, the neurological control of these processes and the immunological network following a dietary modification is still in its early stages of development. Therefore, scientific investigations should aim to explore the influence of nutrients on the regulation of metabolism, epigenetics, and the functioning of the immune system within the autonomous nervous system in order to improve our understanding of the potential advantages of nutritional supplements for neurological disorders [216].

## 11. Guidelines for Integrating Physical Activity into Neurological Treatment Plans

Growing evidence in neuroscience and rehabilitation medicine underscores the critical importance of physical exercise in the treatment of neurological disorders. This review examines guidelines for implementing exercise programs in patients with neurological conditions, focusing on neuroplasticity, the therapeutic benefits of exercise, and specific safety and adaptation considerations for exercise programs. Clinical applications in diseases such as Parkinson’s disease, multiple sclerosis, and recovery after stroke, with an emphasis on personalized treatment, the evidence behind specific interventions, and the need for a multidisciplinary approach, have been discussed.

Neurological disorders, characterized by a wide range of dysfunctions of the nervous system, pose significant challenges for both patients and health care systems. The integration of physical activity into neurological treatment plans is based on a robust understanding of the neurobiological benefits of exercise, including its ability to promote neuroplasticity, improve motor and cognitive function, and facilitate neurological recovery. Physical exercise induces beneficial neuroplastic changes in the brain, facilitating recovery and rehabilitation in neurological disorders. Neuroplasticity, mediated by factors such as increased expression of BDNF, plays a crucial role in the repair and reorganization of the brain following injury. Additionally, exercise improves cerebral circulation, which can contribute to better cognitive function and a reduction in symptoms in neurodegenerative disorders and after stroke [217].

Regarding the guidelines for exercise implementation, before initiating an exercise program, a comprehensive assessment of the patient is essential, including their neurological condition, physical capability, and personal goals. This assessment should inform the selection of exercises and the intensity of the program, ensuring it is safe and effective [218]. Exercise programs should be designed to address the specific rehabilitation goals of the patient, such as improving muscle strength, coordination, or cognitive function. The selection of exercises should be personalized, considering the disease, disease stage, and individual capabilities of the patient [219]. Numerous research initiatives have focused on specific physical activity interventions as a form of neurological treatment. In addition to physical activity benefits, there is evidence suggesting that exercise may also offer health improvements and potentially disease-modifying effects for patients with Parkinson’s disease. This points to the need for clinical trials to further explore these benefits and address safety concerns, such as the increased risk of falls and cardiovascular complications, highlighting the importance of tailored programs that consider potential barriers to improve the quality of life of individuals with neurological disorders [220]. Collectively, these studies affirm the significance of including elderly patients in intervention programs tailored to their specific needs, from preventing falls to addressing broader neurological and physiological vulnerabilities. Similarly, for Parkinson’s disease, there is evidence that exercise regimes emphasizing balance, flexibility, yoga, or tai chi can offer considerable benefits [221]. Similarly, strength training exercises have been shown to significantly improve mobility and movement control, reducing the likelihood of falls.

Physical activity has also shown to be beneficial for multiple sclerosis treatment [222], where exercise protocols incorporate muscle strength training, endurance exercises with fatigue management, and patient-condition-adapted exercises depending on the severity of the disease [219]. The treatment of fibromyalgia through exercise has been proven effective in various studies, offering significant benefits in reducing stress, fatigue, symptoms of depression and anxiety, as well as decreasing pain and insomnia issues. Aquatic exercise has been identified as an effective and complementary alternative to conventional therapy, improving symptoms and the quality of life of patients with fibromyalgia [223]. Additionally, the incorporation of strength training, functional training, aquatic activities, and dance, alongside nutritional advice, is recommended [224]. Evidence also supports the benefits of aerobic exercise and muscle conditioning in these patients, although it is noted that high-quality evidence to support the inclusion of flexibility and balance training is still lacking [225]. Therefore, it is crucial to promote a therapeutic approach that includes physical exercise as a key tool, adapting it to the individual needs and capabilities of the patient to ensure its effectiveness and adherence to treatment.

There is a wealth of evidence supporting the inclusion of elderly patients in intervention programs, particularly for those experiencing neurological issues exacerbated by age-related declines. These patients are especially vulnerable due to a combination of neurological impairments and physiological factors. Intervention protocols tailored to this demographic range from balance and strength exercises to intrahospital interventions prescribed by medical professionals. Similar research showed the significant impact falls have on the elderly, emphasizing [217] the need for a comprehensive understanding of falling causes to devise effective prevention strategies. This underscores the critical nature of including elderly patients in programs designed to mitigate such risks through targeted exercises and interventions [226].

Furthermore, recent studies showed the efficacy of personalized training regimens, which may include balance-training exercises, force platforms, and even tai chi, in reducing falls among ambulatory older individuals [227]. These findings are crucial for developing intervention programs that effectively address the unique needs of the elderly population, particularly those with neurological concerns.

Nevertheless, it is advised that all programs should emphasize the customization of the training regimen to enhance the patient’s motor skills and functional independence. Techniques employed should aim at neuromuscular improvement, with a significant focus on improving gait functionality. In all cases, security is a priority in the implementation of exercise programs for patients with neurological disorders. It is crucial to monitor patients during exercise to prevent injuries and ensure that exercises are performed correctly. Adaptations of exercises and the intensity of the program may be necessary to reflect changes in the patient’s condition [2]. The integration of physical activity into the treatment of neurological disorders requires careful and personalized attention. Evidence suggests that exercise not only improves physical outcomes but also contributes to mental health and the overall well-being of patients. However, variability in individual responses to exercise and the need for treatment-specific adaptations highlight the importance of a multidisciplinary and personalized approach.

## 12. Sports Psychology and Its Role in Neurological Rehabilitation

Neurological rehabilitation is a specialized field within healthcare that focuses on enhancing the physical, cognitive, and emotional well-being of individuals with adverse neurological conditions [228]. Conversely, sports psychology is concerned with enhancing the performance and mental well-being of athletes [228]. Although these two fields may appear distinct, there is a growing recognition of the potential benefits of integrating the principles and techniques of sports psychology into neurological rehabilitation programs. The role of psychology in neurological rehabilitation, particularly within sports, is multifaceted, focusing on the psychological impacts of injuries and the rehabilitation process. Psychology plays a crucial role in understanding and enhancing the mental and emotional well-being of athletes undergoing neurological rehabilitation.

Sports psychology is a discipline that has long been associated with optimizing athletic performance [229]. It includes a range of psychological strategies aimed at improving mental resilience, focus, motivation, and the overall well-being of athletes. These strategies have been proven effective in helping athletes perform at their best in high-pressure situations. Athletes often face setbacks and failures, requiring mental resilience to recover. Similarly, individuals in neurological rehabilitation encounter challenges and frustrations. Sports psychology teaches coping strategies that can help patients navigate these difficulties. Techniques such as mindfulness, stress management, and positive self-talk can empower individuals to maintain a positive outlook and persevere through tough times [230]. Favorable effects have also been observed with the application of positive visualization, a common technique in sports psychology where athletes mentally rehearse successful performances. In neurological rehabilitation, this technique can be adapted to help patients visualize their progress and envision a future where they have regained function and independence. This positive imagery can increase motivation and self-confidence, critical factors in the rehabilitation journey [231].

In sports psychology, setting clear and achievable goals is a fundamental practice. Similarly, in neurological rehabilitation, individuals can benefit from setting specific rehabilitation goals. These goals provide motivation, a sense of purpose, and a plan for progress. Rehabilitation professionals can work with patients to define short- and long-term goals, ensuring they align with the individual’s aspirations and expectations [232]. Another key point is the work on concentration, which is essential for both athletes and rehabilitation patients. Sports psychology offers strategies to improve focus, which can be applied during rehabilitation exercises and therapy sessions. Improved concentration can lead to more effective and efficient rehabilitation [230]. We also see psychological applications in sports where social interactions occur. Teamwork and social support are integral components of sports psychology. Athletes often rely on their coaches, teammates, and support networks for success. In neurological rehabilitation, a similar support system is crucial. Family members, friends, therapists, and support groups play a vital role in providing encouragement and motivation [230].

Another key point in social interactions is the management of achievements and failures. In sports, athletes celebrate their achievements, both large and small, as well as manage failure. This practice can be translated into neurological rehabilitation. Recognizing and celebrating milestones and progress can boost morale and provide positive reinforcement [230]. Beyond the pursuit of goals and social alterations in sports, performance and injury treatment are key to achieving those goals, both being crucial points for neurological treatment. Recent research lines discuss the crucial role of cognitive psychology in sports, focusing on cognitive training, assessment, and rehabilitation. They analyze the effectiveness of cognitive strategies in improving performance and athletic resilience, as well as the future directions for the applications of cognitive psychology in sports. This is relevant for neurological rehabilitation since cognitive aspects are crucial for recovery and performance optimization [233].

Novel therapies such as the relationship between sound and movement in sports and rehabilitation emphasize the importance of understanding the interaction between movement and sound, reviewing the effects of natural movement sounds, movement sonification, and rhythmic auditory information in sports and motor rehabilitation. This approach can aid in the neurological rehabilitation process by enhancing motor learning and motivation through auditory feedback [234]. Despite evidence of athlete improvement with psychological intervention, there are difficulties and reluctances in its application. Recent studies have highlighted the importance of psychological factors in injury treatment, but there is a knowledge and application gap among other specialists despite observations that athletes are psychologically affected 100% of the time when injured [235].

Similarly, the use of basic psychological techniques during the sports injury rehabilitation process has been reported, where athletes expressed interest in receiving more training on advanced sports psychology techniques. This underscores the recognition of psychological aspects in the rehabilitation of sports injuries and the desire for greater integration of these techniques into practice [236]. The multidisciplinary work goes further, and recently the importance of integrating sports psychology and nutrition into an interdisciplinary approach for the rehabilitation of musculoskeletal injuries in professional soccer has been discussed. Incorporating psychological and nutritional components can improve return-to-play models based on sports medicine, highlighting the broader application of psychology in sports rehabilitation [236]. The convergence of sports psychology with neurological rehabilitation illustrates a comprehensive approach that considers recovery as a process involving the body, mind, and spirit. This neurological approach not only enhances physical recovery but also strengthens mental resilience, motivation, and emotional well-being of athletes in the rehabilitation process [237].

## 13. Case Studies and Community-Based Programs of Exercise and Nutrition Interventions in Neurology

Recognizing the paramount role of case studies and their practical application in community-based programs in situating exercise and nutrition interventions within authentic, real-world scenarios is foundational to understanding their true impact. For example, a previous community program named “Start-to-Run” analyzed the impact of a 12-week individualized training program, with a frequency of 3 days per week, conducted within the community. The program aimed to prepare individuals with multiple sclerosis for participation in a running event. The study revealed improvements in aerobic capacity, functional mobility, visuospatial memory, fatigue, and quality of life, along with an increase in pallidum volume among the participants [238]. Similarly, another community-based program utilizing a mobile application, namely, Walk With Me, for individuals with multiple sclerosis demonstrated enhancements in self-reported physical activity, lower limb functional strength, hand function, and cognitive factors following a ten-week individualized walking program guided by the app. The authors also proposed that mobile applications could offer a relatively low-cost and enjoyable distance physical rehabilitation intervention, potentially influencing long-term physical activity behavior in the multiple sclerosis population [239].

In the same way, community-based multimodal exercise has been applied to other neurological disorders. For instance, in Parkinson’s disease, a weekly group session involving circuit-based training for one hour, targeting a perceived exertion effort level of 13 on the 6–20 Borg Scale, demonstrated improvements in physical and cognitive function [240]. Moreover, for individuals who have experienced a stroke, a 12-week home- and community-based exercise program was implemented. This program was tailored individually, considering participant preferences for activities, encompassing any prestroke activities participants wished to resume or work toward resuming, their physical capacity for specific activities, and access to resources in their home and community, including pools, gyms, exercise classes, and therapy programs. The intervention aimed to enhance daily physical activity and reduce sedentary time, adopting a whole-day approach to fostering increased activity. The intervention resulted in a 16% greater improvement in VO2peak during the 6MWT achieved in the intervention group compared to the control group [241]. Finally, community-based exercise programs have also been implemented in individuals with traumatic brain injury, yielding beneficial health and neurological effects [242].

Most of the programs implemented in real-world settings involve evidence-based healthy lifestyle interventions utilizing a multifaceted approach. These interventions encompass physical activity/exercise, dietary and nutrition strategies, and educational components. Nutrition behavior holds particular significance in certain neurological diseases, as malnutrition poses a common risk due to feeding difficulties, therapeutic interventions, and challenges in communication or self-care for dietary needs [243]. Case studies delve into innovative dietary strategies for neurological disorders. For instance, a recent study explored the effects of a 24-week ketogenic diet (comprising 70% fats, 25% protein, and 5% carbohydrates) on symptoms, biomarkers, depression, and anxiety in Parkinson’s Disease [244]. This case study reported improvements in health biomarkers, weight loss, a reduction in cardiac risk factors, and alleviation of anxiety and depression symptoms. Similarly, another case study examined the impact of a ketogenic diet over 18 months on the Expanded Disability Status Scale (EDSS), biomarkers, and walking ability in a man with multiple sclerosis. The study observed improvements in both EDSS scores and walking performance. Consequently, the ketogenic diet emerges as a potential therapeutic approach for neurological diseases such as Parkinson’s and multiple sclerosis [245].

Nutrition combined with physical activity is another crucial factor in fostering general well-being. Participating in physical activity and maintaining a healthy diet were linked to a reduced mortality risk for all causes of death in persons with Parkinson’s disease. A randomized controlled trial carried out by Ngandu et al. in an intervention involving multiple domains showed that older persons at risk of dementia experienced enhanced cognitive performance by adhering to a Mediterranean diet, engaging in physical exercise, and participating in cognitive training [246]. Moreover, Shannon and colleagues had positive results when investigating the feasibility, acceptability, and mechanism of action of a multidomain intervention that focuses on the Mediterranean diet and Physical Activity (PA) for reducing the risk of dementia in a high-risk population [247,248]. Hsieh et al. highlighted in their randomized controlled trial that home-based exercise and nutrition therapies specifically designed for pre-frail or frail older people suffering for cognitive decline can effectively enhance their frailty score and physical performance [249]. Engaging in consistent physical activity and consuming a diet that is abundant in the aforementioned components might decelerate the advancement of the disease in individuals afflicted with neurological diseases, as well as diminish the likelihood of developing the condition in individuals who are in good health. While there is currently limited empirical data from clinical trials regarding the effectiveness of exercise and diet together, it is nevertheless worthwhile to make these efforts. Currently, the most effective non-invasive therapy for a patient involves preparing individualized therapy that focuses on exercise and food. Additional efforts should be made to develop a holistic treatment approach for patients that is both complimentary and inclusive.

In conclusion, the comprehensive examination of various case studies and community-based programs underscores the pivotal role of exercise and nutrition interventions in promoting neurological health and facilitating recovery. The evidence presented showcases the diverse positive impacts on conditions such as multiple sclerosis, Parkinson’s disease, stroke, and traumatic brain injury, emphasizing the adaptability and effectiveness of these interventions across a spectrum of neurological disorders. These findings underscore the importance of integrating tailored exercise programs, community-based initiatives, and innovative dietary strategies into holistic approaches for neurological rehabilitation. As we navigate the intricate landscape of neurological health, future research should persist in unraveling the nuanced interactions between exercise, nutrition, and neurological well-being. The continual exploration and refinement of these interventions are crucial to advancing our understanding and optimizing their application, ultimately contributing to improved outcomes and enhanced quality of life for individuals facing neurological challenges.

## 14. Practical Applications

Tailoring interventions in sports and nutrition for individuals afflicted with neurological conditions represents a pivotal endeavor aimed at augmenting recovery trajectories and optimizing health outcomes. This scholarly pursuit encompasses diverse methodologies and strategies, ranging from the implementation of omega-3 fatty acid-rich diets and adherence to Mediterranean dietary patterns to foster neuroplasticity and enhance cognitive function.

The integration of wearable technology emerges as a crucial facet in this academic discourse, facilitating real-time monitoring of physiological parameters and enabling a meticulous assessment of the impact of exercise regimens on neurological health. Furthermore, the application of neuroimaging techniques assumes significance in unraveling the intricate interplay between dietary and exercise interventions and their consequential effects on both the structure and function of the human brain.

The synthesis of exercise programs, strategically emphasizing elements such as balance, flexibility, and strength training, into the treatment plans for neurological disorders, reflects a sophisticated and comprehensive approach. Complementary to this, the development of targeted nutritional supplements and diets addresses specific facets of neurodegenerative diseases, addressing oxidative stress, neuroinflammation, and micronutrient deficiencies with a precision-driven focus.

Within this scholarly exploration, the investigation of the symbiotic relationship between gut health and neurological well-being unfolds, with an emphasis on the potential contributions of prebiotics and probiotics. Advocacy for personalized exercise and nutrition plans predicated on individual assessments of neurological conditions, physical capabilities, and personal goals underscores the commitment to tailoring interventions to the unique needs of each individual.

The academic endeavor further extends to the exploration of cutting-edge technologies, including virtual reality and immersive technologies, in the realm of rehabilitation exercises. Lastly, the promotion of multidisciplinary approaches, harmonizing physical activity, nutrition, and clinical treatments, emerges as a central tenet in the pursuit of comprehensive neurological care. In essence, the scholarly narrative herein posits itself as a mosaic of interdisciplinary strategies, diligently orchestrated to advance the understanding and enhancement of neurological well-being.

## 15. Methodological Limitations and Potential Biases

A critical examination of the existing research reveals notable methodological limitations and potential biases, which necessitate a cautious interpretation of the findings. Many studies within this domain employ observational designs or are characterized by small, homogenous populations. This limitation inherently restricts our capacity to establish strong causal relationships, particularly concerning the neuroprotective effects of dietary components such as omega-3 fatty acids and adherence to Mediterranean diet patterns. The frequent reliance on self-reported dietary and physical activity data further compounds this issue, introducing recall bias that may distort the findings. To mitigate these concerns, future research endeavors must prioritize experimental rigor and control, enhancing the reliability and validity of the conclusions drawn.

### 15.1. Generalizability and Applicability

The transferability of research findings to the broader population remains a significant challenge, underscored by the inherent diversity in genetic makeup, dietary habits, and lifestyle factors across different demographics. The results derived from specific cohorts, therefore, may not universally apply, underscoring the imperative need for studies that embrace a wider spectrum of participants. Expanding the research framework to encompass varied populations will invariably enrich the generalizability and applicability of the findings, ensuring that the derived insights are reflective of a broader, more inclusive, global context.

### 15.2. Conflicting Evidence

Our review process has unearthed inconsistencies within the literature, especially regarding the effects of physical exercise on cognitive functions amidst neurodegenerative conditions. While there is a substantial corpus of evidence championing the cognitive benefits of consistent physical activity, a fraction of the research presents contradicting outcomes, indicating minimal or null effects. It is plausible that such discrepancies stem from variances in study design, the nature and intensity of the exercise regimen, and the baseline characteristics of participants. This dichotomy not only highlights the intricate biological intricacies at play but also emphasizes the importance of adopting personalized approaches in both the investigative and therapeutic realms.

### 15.3. Future Research Directions

Conducting longitudinal studies represents a foundational initiative for the systematic examination of enduring ramifications arising from specific dietary patterns on the progression of neurodegenerative diseases. Simultaneously, rigorous clinical trials are imperative for elucidating the efficacy of novel neuroprotective nutrients and supplements in fostering neurological recovery, thereby contributing to the scientific understanding of therapeutic interventions.

An intricate exploration into the mechanisms underpinning the modulation of neuroinflammation and neurogenesis by physical activity and diet constitutes an essential component of scholarly inquiry. Moreover, the scholarly discourse extends to meticulous investigations delving into the nuances of personalized nutrition and exercise interventions tailored to address specific neurological conditions, embodying a commitment to precision-based healthcare approaches.

Integral to this academic pursuit is the exploration of the symbiotic relationship between technology-enhanced physical activity and nutritional monitoring, facilitating real-time adjustments in therapeutic protocols. Furthermore, comprehensive studies scrutinizing the influence of gut microbiota on neurological health and the potential therapeutic benefits arising from dietary interventions illuminate the intersection between nutritional sciences and neurological well-being.

The investigation of intermittent fasting or caloric restriction emerges as a focal point, wherein its impact on neuroplasticity and cognitive function is systematically scrutinized. Pioneering efforts in technological innovation are evident in the development of wearable and implantable devices, facilitating continuous monitoring of physiological responses to dietary and exercise interventions in neurological conditions.

Concurrently, the exploration of the synergistic effects of combined physical and cognitive activities constitutes a noteworthy avenue, aimed at enhancing both cognitive and physical well-being within the context of neurodegenerative diseases. The scholarly narrative further encompasses investigations into the epigenetic changes induced by diet and exercise, offering insights into their implications for neuroprotection and recovery. In essence, this academic discourse underscores a methodical and comprehensive approach to advancing our understanding of the intricate interplay between lifestyle factors and neurological health.

## 16. Conclusions

The synthesis of insights from our comprehensive review underscores the pivotal role of the synergy between sports nutrition and neurological health in enhancing neurological outcomes. This interaction not only plays a fundamental role in the prevention, treatment, and rehabilitation of neurological conditions but also opens avenues for innovative therapeutic strategies. Our findings illuminate the significant contributions of targeted nutritional interventions, such as the incorporation of omega-3 fatty acids and adherence to Mediterranean dietary patterns, towards promoting neuroplasticity and facilitating recovery processes. Additionally, the integration of regular physical activity into lifestyle choices emerges as a cornerstone for mitigating the progression of, and potentially reversing the impacts of, various neurological disorders, attributed to its neuroprotective and cognitive function-enhancing effects.

Emerging technologies, including wearable devices and advanced neuroimaging techniques, are revolutionizing our ability to monitor and understand the complex dynamics at play between physical activity, dietary habits, and neurological health. These technologies not only enhance the precision of our assessments but also enable personalized approaches to treatment and prevention strategies, catering to the unique needs of individuals.

Despite the strides made in understanding these interconnections, our review has identified critical gaps that warrant further investigation. There is a pressing need for longitudinal studies to explore the long-term effects of specific dietary patterns and physical activity regimens on the progression and management of neurodegenerative diseases. Moreover, the mechanisms underlying the modulation of neuroinflammation and neurogenesis through diet and exercise have yet to be fully elucidated. Future research should also aim to develop and evaluate personalized nutrition and exercise interventions, leveraging the potential of emerging technologies for real-time monitoring and intervention adjustments.

The integration of sports psychology into neurological rehabilitation presents another promising frontier. Understanding the psychological aspects of rehabilitation can enhance motivation, adherence, and overall outcomes for individuals with neurological conditions. Exploring the role of community-based programs and case studies can provide practical insights into implementing these interventions effectively in real-world settings.

In conclusion, our review advocates for a multidisciplinary approach, combining insights from neurology, sports science, nutrition, and psychology, to develop holistic strategies for improving neurological health. By addressing the identified research gaps, we can advance our understanding of the interplay between sports nutrition and neurological health, ultimately leading to more effective interventions and improved quality of life for individuals with neurological conditions.

## Figures and Tables

**Table 1:** Most notable clinical trials named where exercise has brought improvements to patients with neurological problems.

Authors	Study Desing	Study Participants	Disorder	Type of Exercise	Results
Yuan et al. [165]	Clinic trial	N = 24. Aged more than 65 years	Parkinson Disease	Interactive video-game-based (IVGB) exercise program for 30 min three times per week the first 6 weeks	Improved the balance, postural stability, and confidence
Kolk et al. [166]	Clinic trial	N = 65 Aged from 30 to 65 years	Parkinson Disease	30–45 min training three times per week for 6 months	Aerobic exercise can be done at home by patients with Parkinson’s disease with mild disease severity and it attenuates off-state motor signs
Brandín de la Cruz et al. [168]	Clinical Trial	N = 12 Aged more than 65 years	Parkinson Disease	12 sessions of 30 min, evenly distributed over a period of four consecutive weeks.	The viability of integrating an antigravity treadmill with an immersive virtual reality system for the rehabilitation of individuals with PD.
Molhemi et al. [170]	Clinic trial	N = 35 Aged from 18 to 64 years	Multiple Sclerosis	35 min training three times per week during 6 consecutive weeks	Both the Virtual Reality-based and conventional balance exercises improved balance and mobility in people with MS
Grazioli et al. [171]	Clinic trial	N = 20 Aged from 25 to 55 years	Multiple Sclerosis	12 weeks combined training intervention (resistance and aerobic exercise)	Improvement in walking and balance ability as well as reduced depression and fatigue
Shimada et al. [162]	Clinic trial	N = 945 Aged more than 65 years	Mild Cognitive Impairment	40 weeks program of combined cognitive and physical activity with those of a health education program	Combined physical and cognitive activity improves or maintains cognitive and physical performance in older adults with mild cognitive impairment
Makino et al. [174]	Clinic trial	N = 415 Aged more than 70 years	Alzheimer Disease	26 weeks of aerobic exercise training, resistance exercise training and combined exercise training	Improve delayed memory in community-dwelling older adults
Inskip et al. [175]	Clinic trial	N = 9 Aged from 66 to 84 years	Lewy Body Dementia	8 weeks three times per week static balance, dynamic balance, functional practice, and progressive resistive exercise.	Improved clinically meaningful amounts in functional independence, cognition, physical function, and strength

**Table 2:** Most relevant studies where nutritional supplements have significant impact on neurological functions.

Authors	Study Desing	Disorder	Supplement	Results
Pacifici et al.’s [199]	In vitro Clinical-Trial	Parkinson’s Disease	A5^+^, a combination of polyphenols and micronutrients	A novel mixture of polyphenol and micronutrients known as A5^+^, by acting in a synergic manner, can counteract the noxious processes
Alizadeh et al. [201]	Case-Control	Parkinson’s Disease	β-carotene, vitamin C, riboflavin, vitamin B6, and biotin	An adequate dietary intake of vitamins and minerals may have a preventive effect on developing PD
Jamali et al. [203]	Clinical-Trial	Parkinson’s Disease	β-carotene	There is a positive effect of β-carotene administration in PD rats
Sachs et al. [207]	Randomized-controlled clinical trial	Mild Cognitive Impairment	Multivitamin-mineral treatment and cocoa supplementation	Over 3 years, 110 incident MCI and 14 incident dementia cases were adjudicated. Incidence rates did not vary by assignment to multivitamin-mineral or cocoa extract
Jia et al. [210]	Clinical-Trial	Alzheimer Disease	Prebiotic chitosan oligosaccharide (COS)	Orally administered COS at 200, 400, or 800 mg/kg doses were effective at reducing the learning and memory deficits in Aβ1-42-induced rats
Gupta et al. [213]	In vitro Clinical-Trial	Alzheimer Disease	ω-3 fatty acid docosahexaenoic acid	Essential ω-3 fatty acid docosahexaenoic acid acts in a dose-dependent manner to prevent the actions of palmitic acid on inflammatory signaling in astrocytes

## Data Availability

Not applicable.
